# On the Accurate Identification of Network Paths Having a Common Bottleneck

**DOI:** 10.1155/2013/890578

**Published:** 2013-11-12

**Authors:** Muhammad Murtaza Yousaf, Michael Welzl

**Affiliations:** ^1^Punjab University College of Information Technology (PUCIT), University of the Punjab, Allama Iqbal (old) Campus, Lahore, Pakistan; ^2^Networks and Distributed Systems Group, Department of Informatics, University of Oslo, Norway

## Abstract

We present a new mechanism for detecting shared bottlenecks between end-to-end paths in a network. Our mechanism, which only needs one-way delays from endpoints as an input, is based on the well-known linear algebraic approach: singular value decomposition (SVD). Clusters of flows which share a bottleneck are extracted from SVD results by applying an outlier detection method. Simulations with varying topologies and different network conditions show the high accuracy of our technique.

## 1. Introduction

End-to-end flows in packet based networks adversely influence each other when they traverse a network link which is a single point of congestion. We call such a link a “shared bottleneck”. Given the large number of distributed applications that are now used on the Internet, where coordination between such end-to-end flows is quite feasible, we believe that the detection of shared bottlenecks in the network is one of the key measurement efforts that can be undertaken, as there are numerous possibilities for using such knowledge.

Consider, for example, a transfer delay prediction tool (which is a common element of computational Grids) which tells two senders A and B that the time to transfer files to receivers C and D will be four and five minutes, respectively, but fails to inform them that the A-C and B-D path share a bottleneck. Then, both systems would perhaps start to send the files at the same time, and the predicted transfer delay would clearly be wrong because of adverse interactions between the flows at the shared bottleneck. In a Grid, it is not uncommon for a single entity (or person) to be in control of the two senders A and B—thus, with knowledge about shared bottlenecks, this problem might have been prevented by accordingly scheduling the time of these transfers (this is what schedulers in Grid workflow systems do).

As an another example, one could imagine an overlay network where a file is sent from a single source to a single destination via multiple paths across the overlay in order to speed up the transfer time. Such a scheme would probably only yield a noticeable benefit if the overlay paths that are used do not include a shared bottleneck in the underlay. Similarly, in peer-to-peer systems, knowledge about shared bottlenecks could theoretically be used for appropriately rearranging the way documents are placed in order to exploit multiple paths or avoid adverse interactions among simultaneously active downloaders.

To the best of our knowledge, despite the existence of some measurement studies on shared bottleneck detection (cf. [1–3]), such endeavors are hardly undertaken. This may be due to problems with the existing measurement methods, such as the amount of data that they need to transfer, or the time that a measurement would take. Most notably, the accuracy exhibited in recent studies is limited—this may be a reason for designers of new network mechanisms to refrain from using such measurement results in practice.

In this paper, we address this issue with a method that only operates on end-to-end measurements (forward delays), is easy to calculate, and provides extraordinarily accurate results. Our scheme is based on a combination of the well-known technique of singular value decomposition (SVD) of principal component analysis (PCA) and outlier detection.

After a brief survey of related work in the next section, we will provide the mathematical foundations of our mechanism in Section 3. The mechanism itself will be explained in Section 4, and results from simulations will be presented in Section 5.  Section 6 concludes with an outlook on future work.

## 2. Related Work

The awareness of the complete network topology and particularly the knowledge of congested network links which are shared by multiple flows can be of great use to many applications. A great deal of work has already been done in this area which directly or indirectly relates to our study. In what follows, we briefly discuss some of the related efforts.

Two techniques have been proposed in [1] which are mainly based on correlation: one is based on the forward delays of the packets and the other relies on packet loss. The decision of whether two flows share the same bottleneck or not is made by a comparison test. Such a test would compare the cross measure (cross-correlation) and automeasure (autocorrelation), so the decision is not only taken on the basis of a single correlation function.

In the delay based technique, the cross measure is computed by merging the delay sequences of the two flows which are to be tested. From this merged sequence, only those pairs of samples are picked in which the first sample of a pair is from one sequence and the second sample of pair is from another sequence. Then, from all such samples, the cross measure is calculated between the samples of the first and the second sequence. The automeasure is calculated as the correlation coefficient between the delays of adjacent packets of a single flow. After the computation of the cross measure and the automeasure, the comparison test declares that the two paths share a bottleneck if the automeasure is less than the cross measure.

In the loss based technique, the cross measure and the automeasure are actually the conditional probabilities that a packet is lost given that the following packet is also lost. Both techniques work in the topologies where the senders or receivers are colocated. It is also shown that the delay based technique attains accuracy quite quickly as compared to the loss based technique.

The entropy based technique presented in [4] works in topologies where some sources are connected to a single destination with the assumption that each path holds only one bottleneck. Passive measurements are used in this technique to measure the interarrival times at the destination. A cluster of flows is formed and the average entropy is calculated for every flow with the assumption that the flow belongs to this cluster. After that the flow is put into the cluster with the minimum average entropy. The clustering correctness of this technique only gives acceptable results in case of low cross traffic and does not scale well with heavy cross traffic.

We consider FlowMate [3] as one of the most relevant works for us. It uses the comparison test for the delay based technique of [1] and works on the online clustering of flows by comparing each new flow with a representative flow of every cluster. Initially, one flow is designated in each cluster and later every new flow is compared with only one representative of each cluster. Another simple clustering mechanism is also discussed in [3] where representatives of each cluster can be switched dynamically. The accuracy of FlowMate, however, becomes a problem in case of short lived HTTP connections because it does not get enough samples.

One of the latest works in this regard is by Kim et al. [5], where a clustering approach which uses multidimensional indexing is presented. This work is based on the previous work presented in [2], where the cross-correlation was used to detect whether two paths share a bottleneck. This cross-correlation is computed between the delay sequences of two paths after applying wavelet denoising on the original delay sequences.

One thing which is common in all the above-mentioned approaches is that all of them operate in a pairwise fashion. All techniques consider two paths to detect if flows share a bottleneck whereas our mechanism works for any number of flows at the same time, resulting in clusters of paths which share a bottleneck.

A loss based technique which expects a common sender to send packet pairs has also been proposed [6]. If the probability of the second packet being lost exceeds a particular threshold, it is assumed that the two paths share a point of congestion. This technique requires DropTail queuing and can suffer from all the problems which exist for packet pair based measurements.

## 3. Multivariate Analysis

Multivariate analysis has most of its applications in image processing and pattern recognition, but the nature of our problem makes multivariate analysis an ideal tool in solving it. We decided to apply multivariate analysis because we believe that there are many factors which are beyond the scope of a simple correlation method. Multivariate analysis is highly suitable in such scenarios because we want to check the principal factors which mean the delays caused by the congested link.

We will see that SVD provides us with highly accurate results in terms of identifying the flows which share a bottleneck. SVD is one out of many multivariate analysis techniques in the literature, and others should eventually also be considered in terms of efficiency and reliability.

We construct a matrix **A** in which the rows are the flows and the columns are time slots. An entry in **A** is the amount of (average) delay measured during experiments. We will analyze this data matrix **A** using SVD.

### 3.1. SVD and Spectral Analysis

We briefly review the singular value decomposition of matrices; we will use some of its properties in our discussion in Section 4.2.

Any **m** × **n** matrix **A** can be expressed as

(1) $$\begin{array}{c} A=\sum_{t=1}^{r}\sigma_{t}\left(A\right)u^{\left(t\right)}v^{{\left(t\right)}^{r}}, \end{array}$$

where *r* is the rank of *A*, *σ*_1_(*A*) ≥ *σ*_2_(*A*) ≥ ⋯*σ*_*r*_(*A*) > 0 are its singular values, and *u* ^(*t*)^ ∈ *ℛ* ^*m*^, *v* ^(*t*)^ ∈ *ℛ* ^*n*^, *t* = 1,…, *r* are its left and right singular vectors, respectively. The *u* ^(*t*)^'s and *v* ^(*t*)^'s are orthonormal sets of vectors; namely, *u* ^(*i*)^*T*^^ *u* ^(*j*)^ is one if *i* = *j* and zero otherwise. We also remind the reader that

(2) ||A||F2=∑i,jAij2=  ∑i=1rσi2(A),||A||F2=max⁡xϵℛn:||x||=1||Ax||=max⁡xϵℛm:||x||=1||xTA||=σ1(A).

In matrix notation, SVD is defined as *A* = *U*Σ*V* ^*T*^ where *U* and *V* are orthogonal (thus *U* ^*T*^ *U* = *I* and *V* ^*T*^ *V* = *I*) matrices of dimensions *m* × *r* and *n* × *r*, respectively, containing the left and right singular vectors of *A*. Σ = diag⁡(*σ*_1_(*A*) ≥ *σ*_2_(*A*) ≥ ⋯*σ*_*r*_(*A*)) is an *r* × *r* diagonal matrix containing the singular values of *A*.

One of numerous applications of SVD is in recovering the structure of matrices and in noise removal in a variety of settings. The underlying idea is very simple: since *A* = ∑_*t*=1_^*r*^ *σ*_*t*_(*A*)*u* ^(*t*)^ *v* ^(*t*)^*r*^^, we can create approximations to *A* by keeping only the top *k* “principal components” (i.e., the top *kσ*_*t*_(*A*)*u* ^(*t*)^ *v* ^(*t*)^*r*^^) for various values of *k*. Essentially, discarding the “smallest” principal components (the ones corresponding to the smallest singular values) results in a small loss in accuracy, and we might justifiably consider those components as “noise”. This procedure is commonly referred to as “principal component analysis,” and the following theorem (usually attributed to Golub and Loan [7]) quantifies the loss of accuracy incurred by keeping only the top *k* components for various values of *k*.

**Theorem 1**

Let *A*_*k*_ = ∑_*k*=1_^*r*^ *σ*_*t*_(*A*)*u* ^(*t*)^ *v* ^(*t*)^*r*^^ (for any 1 ≤ *k* ≤ *r*). *A*_*k*_ is the “best” rank⁡ *k* approximation to *A* with respect to the 2-norm and the Frobenius norm; namely, for any matrix *D* of rank⁡ at most *k*,

(3) $$\begin{array}{c} {||A-A_{K}||}_{2}\leq {||A-D||}_{2},\\ {||A-A_{K}||}_{F}\leq {||A-D||}_{F}. \end{array}$$

Also,

(4) $$\begin{array}{c} {||A-A_{K}||}_{F}^{2}=\sum_{t=k+1}^{r}\sigma_{t}^{2}\left(A\right),\\ {||A-A_{K}||}_{F}\leq \sigma_{k+1}\left(A\right). \end{array}$$

We say that a matrix *A* has a “good” rank⁡  *k* approximation if the 2-norm and the Frobenius norm of *A* − *A*_*K*_ are small; for a detailed treatment of singular value decomposition, see [7].

### 3.2. Intuition

The main intuition behind using SVD based approach was obtained from the work of Camtepe et al. [8] where the authors used SVD based approach to discover hidden communities and communication patterns in a chatroom. A tool was built for the data collection of individual chatters, and then an SVD based algorithm was applied to locate groups of chatters communicating with each other. The authors used the number of messages exchanged during a time window as data and found the groups of chatters communicating with each other on the basis of a similar number of messages exchanged. We mapped this to our problem by comparing different nodes in the network with the chatters in the chatroom and considering forward delays along a path as data entries during a time window, assuming that this should provide us with an association among the paths with similar delay patterns during certain periods of time. As the end-to-end delay along a network path is mainly influenced by the bottleneck, we assumed that SVD must cluster the paths with similar delay patterns during a certain period of time or, in other words, that share the same bottleneck.

## 4. SVD Based Shared Bottleneck Detection

A bottleneck is a network link/router where the packets of a flow experience excessive delays or they are even lost. Most of the techniques proposed till now to detect shared bottlenecks are based on packet delays or packet loss. It has been shown that delay based techniques quickly converge towards accurate results [1]; hence, we decided to rely on the same metric in our mechanism, where one-way delays along a path are used to detect shared bottlenecks. The whole approach is summarized in Algorithm 1. We can divide it into three major steps:

1. measurement of one-way delays along all the paths and obtaining a covariance matrix,
2. applying SVD on the covariance matrix and getting a projection,
3. grouping paths that share a bottleneck.

### 4.1. Delay Measurements

First of all we measure the one-way delays for all the paths by initiating a flow on every path. We group samples of delay measurements on the basis of time windows and pick the average of a time window as a representative delay measurement for that particular time window. We have worked with different time windows and observed no significant change in our results; this is explained in Section 5.2(3). After checking with different window sizes, we decided to use 0.3 seconds as an optimal value for realistic network conditions.

Clearly, this value is a tradeoff between accuracy (the smaller the window, the more information we have) and overhead (we need to collect the necessary data at the network node that will carry out the calculation). We can also reduce or expand the time window size depending on the delay samples available; for instance, in case of short lived flows, we can reduce it, and, in case of long lasting flows, we can expand it, for example, if we need to work in a fully passive environment. At the end we form a matrix *D* of forward delays of all flows as shown in (5) where each row represents the delays experienced by a single flow. Each entry *d*_*ij*_ is an average delay measurement of the *i*th path for the *j*th time window:

(5) $$\begin{array}{c} D=\left[\begin{array}{ccc} d_{11} & \cdots & d_{1n}\\ \vdots & \ddots & \vdots \\ d_{m1} & \cdots & d_{mn} \end{array}\right]. \end{array}$$

The variability of the delays experienced by all flows can be summarized by computing the covariance matrix *A* of the delay matrix *D*. Given an *m* × *n* delay matrix *D*, whose *m* rows represent different paths and whose *n* columns have average delay measurements, the covariance matrix of *D* is the matrix *A* which has entries *a*_*ij*_ as defined in (6):

(6) $$\begin{array}{c} a_{ij}=\text{covariance}\left(d_{i*},d_{j*}\right). \end{array}$$

In short, *a*_*ij*_ is the covariance of delay measurements of the *i*th and *j*th path in the delay matrix. The covariance of two sets of data is a measure of how strongly they vary together. When *i* = *j*, then the covariance is the variance of the delay measurements of a path. 

Here, it is important to note that, if clocks are not synchronized between sender and receiver, then all the delay measurements will be shifted by a certain amount. The shifting will not affect the result of SVD based approach because the important factor for the result is the delay pattern and not the magnitude of actual delays, so the lack of time synchronization will only cause a shift in actual delay values, but the delay pattern will still be preserved. 

### 4.2. Applying SVD and Projecting

Having the covariance matrix *A*_*m*×*m*_ of delay measurements, we apply SVD on *A*_*m*×*m*_ to get singular values of our delay measurements for all the paths. As a result we get the left singular matrix *U*_*m*×*r*_, the diagonal matrix Σ_*r*×*r*_, and the right singular matrix *V*_*n*×*r*_. Here, the matrix *U*_*m*×*r*_ represents the association among the paths on the basis of similar delays caused by common bottlenecks, and the matrix Σ_*r*×*r*_ represents the degree of this association. Then, on the basis of rank reduction of the diagonal matrix Σ_*r*×*r*_, we project the matrix *U*_*m*×*r*_ on the *c* column(s) (for  any  1 ≤ *c* ≤ *r*) of the diagonal matrix to look for *c* dimensions. The value of *c* can be decided by looking at the explained variance of the elements of the diagonal matrix. For instance, if the first element of the diagonal matrix is explaining the data 60%, then we may need to take the second or third dimensions. If the first one explains 90% of the data, we say there is rank reduction and project on the first column of the diagonal matrix to take only the first dimension.

Usually, if the paths under investigation share a bottleneck, then the diagonal matrix always shows a good rank reduction and we need to project only on the first column of diagonal matrix. In order to project matrix *U*_*m*×*r*_ on the *c*th column of the diagonal matrix, we multiply each row of *U*_*m*×*r*_ with only the *c*th column of the diagonal matrix. This results in a matrix *X*_*m*×1_, as shown in (7), that has one entry corresponding to each path, and these values are further used to group flows on the basis of shared bottlenecks:

(7) $$\begin{array}{c} X=\left[\begin{array}{c} x_{1}\\ \vdots \\ x_{m} \end{array}\right]=\left[\begin{array}{ccc} u_{11} & \cdots & d_{1r}\\ \vdots & \ddots & \vdots \\ u_{m1} & \cdots & d_{mr} \end{array}\right]\left[\begin{array}{ccc} \sigma_{11} & \cdots & 0\\ \vdots & \ddots & \vdots \\ 0 & \cdots & \sigma_{rr} \end{array}\right]. \end{array}$$

### 4.3. Grouping Flows

**Figure 1:**
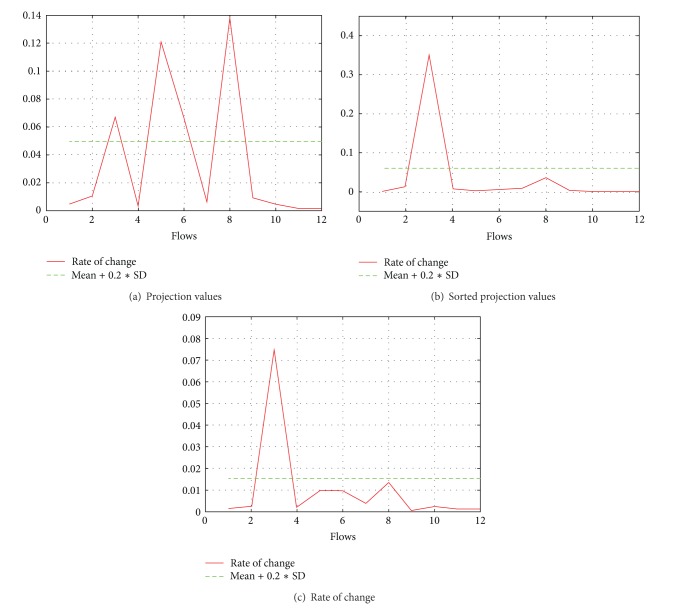
Results in intermediate and final form.

As already stated, the matrix *X*_*m*×1_ contains one value for each path. Here, the values for paths that share a bottleneck are similar, and they differ significantly from the values of other paths. A simple plot of such values will look as in Figure 1(a). If we sort all the entries of matrix *X*_*m*×1_ in a descending order and plot the sorted data, then even at this step we can easily visualize some clusters as exhibited for the same data in Figure 1(b), which shows our result of one of the experiments with 25 paths and 3 bottlenecks. If we visualize the data as a graph, we can observe some clear grouping on the basis of stable slopes and sudden drops in the slope indicating the start of a new cluster. 

We find the rate of change (roc) between two successive values of the sorted list by using

(8) $$\begin{array}{c} \text{roc}=\frac{x_{i}-x_{i+1}}{x_{i+1}}. \end{array}$$

The rate of change could be calculated by dividing the successive differences with the denominator being either *x*_*i*_ or *x*_*i*+1_. As the values have already been sorted in a descending order, it is obvious that this series exposes the break points better if we choose *x*_*i*+1_ as a denominator as compared to *x*_*i*_ which is shown in Figure 1(c). Once we have this series of rate of change, the next step is to detect the break points which becomes a simple problem of detecting outliers.

In order to detect outliers, we used the threshold *τ* defined as

(9) $$\begin{array}{c} \tau ⟵\overset{-}{z}+0.2s, \end{array}$$

where $\overset{-}{z}$ is the mean rate of change and *s* is its standard deviation. The coefficient value of 0.2 was selected after a detailed study of data obtained in simulations. As we will see in Section 5, our method works very well with this value in a wide range of network conditions and scenarios.

### 4.4. Time Complexity of the Whole Algorithm

It is important to have a computable time complexity for situations where a mechanism needs to be applied in real time. In this section, we discuss the time complexity of our algorithm and we show that it is feasible to use online. It has three major parts. During the first part, we extract singular values from delay measurements, then in the second phase we find the projection of left singular vectors over the first singular value, and finally in the third and last phase we identify clusters of paths that share a bottleneck.

First of all let us discuss the time complexity of SVD which is *O*(*mn* ^2^) for a matrix of order *m* × *n* [7]. Here, the dominant part is the number of columns of a matrix. In our technique, the number of columns or *n* is the number of delay measurements used to detect shared bottlenecks. In Section 5, we will show that very few measurements (1 measurement is the average of all samples taken in 300 milliseconds) are enough to obtain an accurate result. The time complexity of *O*(*mn* ^2^) is not expensive at all when we have an upper bound on *n*, so if we consider *n* as a constant, then the complexity becomes *O*(*m*), which means that it depends only on the number of paths under investigation. It is also true if covariance matrix is used instead of simple delay measurement matrix.

In an online version, we may need to update our results in a continuous fashion because the bottleneck link may vary depending on the load of network traffic. Solving SVD for one matrix of delay measurements allows for an incremental, efficient solution for letting the matrix slide over in time (i.e., throwing away the oldest measurements and bringing in the most recent). In such situations, the time complexity of SVD is further reduced to *O*(*mk*) [9] where *k* is the size of the data set.

The second phase involves the projection which mainly multiplies the first element of each row of matrix *U* with the first element of the first column of matrix Σ. This procedure takes *m* multiplications which means a time complexity of *O*(*m*).

We get *m* values corresponding to each path as a result of second stage. In the final part of our procedure, we first sort these values, which may take *O*(*m*log⁡⁡*m*) steps (e.g., if quicksort is used). At the end, for grouping paths which share a common bottleneck, we need to compare the sorted list with a threshold. This can be done in *O*(*m*) steps because it just involves *m* comparisons. We perform all above steps in serial so the most dominant part defines the time complexity of our whole algorithm. The total time complexity is, therefore, *O*(*m*log⁡⁡*m*) where *m* is the number of paths to be inspected for common bottlenecks. This shows that it is practical to use this approach in real time.

## 5. Performance Evaluation

In this section, we present the results of using our mechanism in simulations. We used the ns-2 [10] network simulator to examine different scenarios. We have applied the same simulation topologies and parameters which were investigated in [3] because we consider it as one of the most relevant works to us. One of the two topologies used in [3] is simple and symmetric, while the other one is more complex and asymmetric. As in both topologies the link delays after bottlenecks are similar, a delay matrix was used to obtain SVD results for all experiments. The only exceptions are the experiments discussed in Section 5.2(8), where a topology is used which has longer delays at some branches after the bottlenecks. There, it will be shown that the use of the delay matrix for SVD shows its limitations in such situations, and hence the appropriate choice is the covariance matrix which could be used in all cases. 

**Figure 2:**
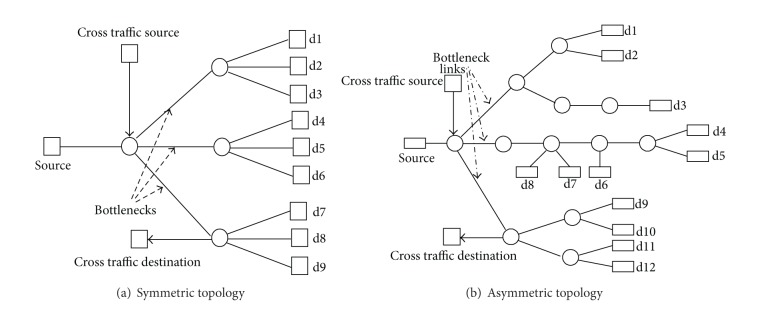
Symmetric and asymmetric topologies.

The symmetric topology is shown in Figure 2(a). It contains three different bottlenecks on three different branches of paths. Each branch has three different destinations for a common source. All the links capacities are 10 Mbps except for the bottleneck links of the first two branches. The bottleneck links at the first two branches have a capacity of 1.5 Mbps and 3.0 Mbps, respectively, whereas the third bottleneck is created by heavy cross traffic from a number of constant bit rate (CBR) flows. Background traffic is generated in both directions from source to destinations by a number of multiplexed Pareto flows. The rest of the simulation parameters are summarized in Table 1.

The asymmetric topology is shown in Figure 2(b). It also has three different branches with a bottleneck at each branch. The mechanism for creating the bottlenecks is the same as for the symmetric topology. It has a varying number of destinations, and, for each destination, it has a varying number of links for the end-to-end path. Simulation parameters are given in Table 1 except for the number of foreground TCP flows which is 12–36.

### 5.1. Performance Metrics

Three different performance metrics which can be considered for measuring the accuracy have been used in [2] as follows.

 (i) *False Positive*: a flow does not share a bottleneck, but it is considered as being part of a group that shares a bottleneck.

(ii) *False Negative*: a flow shares a bottleneck with a group but is not identified as part of the group. 

(iii) *Correct Clustering*: a flow is in the same group as flows with which it shares a bottleneck.

The accuracy index (AI) presented in [3] takes all of the above performance metrics into account and measures the accuracy of clustering in a unified manner. If there are *N* paths, *P*_*c*_ is the set of correct clusters, *P*_*o*_ is the set of clusters as a result, *n*_*fs*_ is the number of flows erroneously included in a resulting cluster, and *s*_*j*_ is the number of subclusters of a correct cluster that was split into *s*_*j*_ subclusters in *P*_*o*_, then AI is calculated as

(10) $$\begin{array}{c} \text{accuracy}\;\;\text{index}\left(\text{AI}\right)=1-\frac{\sum_{i=1}^{\left|P_{o}\right|}{\left(n_{fs}\right)}_{i}}{N}-\frac{\sum_{j=1}^{\left|P_{c}\right|}\left(s_{j}-\;1\right)}{N}. \end{array}$$

### 5.2. Results

In this section, we discuss the results of our experiments in different ways to highlight the accuracy of our SVD-based method for detecting shared bottlenecks.

(*1) Symmetric Topology*. To illustrate our whole mechanism, we first used the symmetric topology shown in Figure 2(a) with 9 TCP flows (one for each destination). In this experiment, flows 1–3, 4–6, and 7–9 each share a bottleneck. For better understanding, in the first row of Table 2, we show the values of the diagonal matrix Σ which exhibits a nice rank reduction, whereas in the second row the values of matrix *V* are shown; these are the results after projection of the left singular matrix *U* on the first component of the diagonal matrix Σ (as explained in Section 4.2). Just from a brief look at the values of *V* in Table 2, we can visualize a grouping of flows 1–3, 4–6, and 7–9.

**Figure 3:**
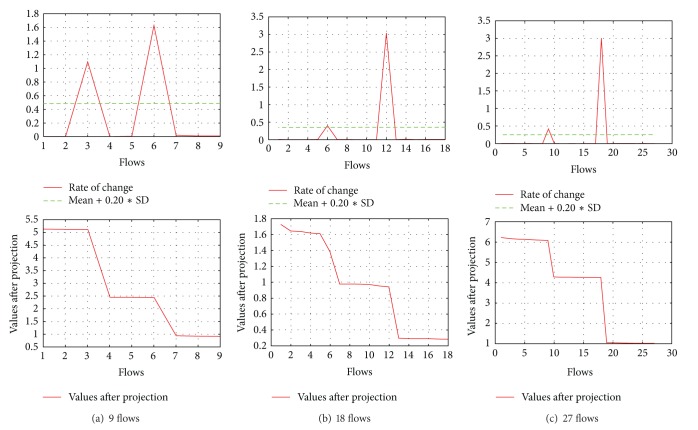
Results of symmetric topology with varying number of flows.

As the next and final step, we calculate the rate of change for the values of matrix *V* as explained in Section 4.3 and find the outliers to detect the group of flows which share a bottleneck. The results are shown together with the absolute values of *V* in Figure 3(a); they are accurate according to the simulation scenario.

**Figure 4:**
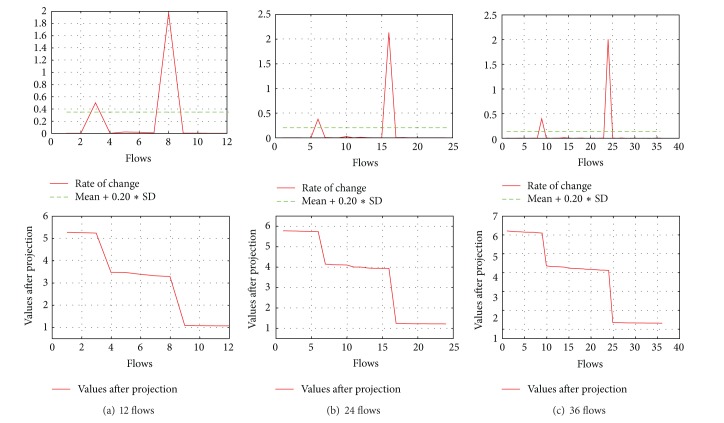
Results of asymmetric topology with varying number of flows.

We conducted the same experiment with 18 and 27 foreground TCP flows to check the effect of multiple TCP flows over the same bottleneck. In these experiments we started 2 and 3 TCP flows for the same destination, using a random start time within the first second of the simulation in order to minimize phase effects [11]. In both experiments, we obtained 100% accurate shared bottleneck recognition (i.e., accuracy  index  (AI) = 1). The results are depicted in Figure 3.

(*2) Asymmetric Topology*. We performed experiments with 12, 24, and 36 flows with random start time within the first second of the simulation using the asymmetric topology and found AI to be always 1. The results are shown in Figure 4.

(*3) Window Size.* We used the asymmetric topology with 12 foreground TCP flows (representing 12 paths). In this experiment, we wanted to check the impact of the window size on the accuracy of our method. By window size we mean the time interval for which we combine all delay measurements and choose one representative which is the average of that interval. A window size of 0.3 seconds is working fine, in what follows; we justify the use of this value. We ran the simulation for 60 seconds and obtained results after setting different window sizes. It is possible that, if we keep on reducing the size of the window, we will reach a point when some windows may not have even a single measurement due to congestion, meaning we will not have even a single delay measurement for that particular window. This must be avoided because the SVD may result in inaccurate results if we use some blanks or imaginary values in the matrix of delay measurements. At the same time, we should not make the window size too large because this could blur the information about the correct bottleneck, and the time series of delay measurements of different flows may not show any association.

During this experiment we varied the window size from 0.1 seconds to 1.0 seconds, increasing it by 0.1 seconds. The resulting accuracy index was 1 after a window size of 0.2 onwards, as shown in Figure 6(a).  Figure 6(b) shows very stable slopes with significant rate of change (roc) for projected values for all the windows sizes we investigated. This makes it much easier to identify cluster of flows that share a bottleneck.

As already mentioned, we started from a very small window size of 0.1. In this case, there were many windows for which we could not have even a single delay measurement. We placed −1 in the empty windows because SVD does not work with empty cells in the matrix. We show the percentage of negative values (i.e., empty windows) against the window size in Figure 6(c). We notice that the percentage of empty cells for a window size of 0.2 is 2.41%, but even then we got accurate results as depicted in Figure 6(a). This means that our method is strong enough to cope with some percentage of missing data less than or equal to 2%.

**Figure 5:**
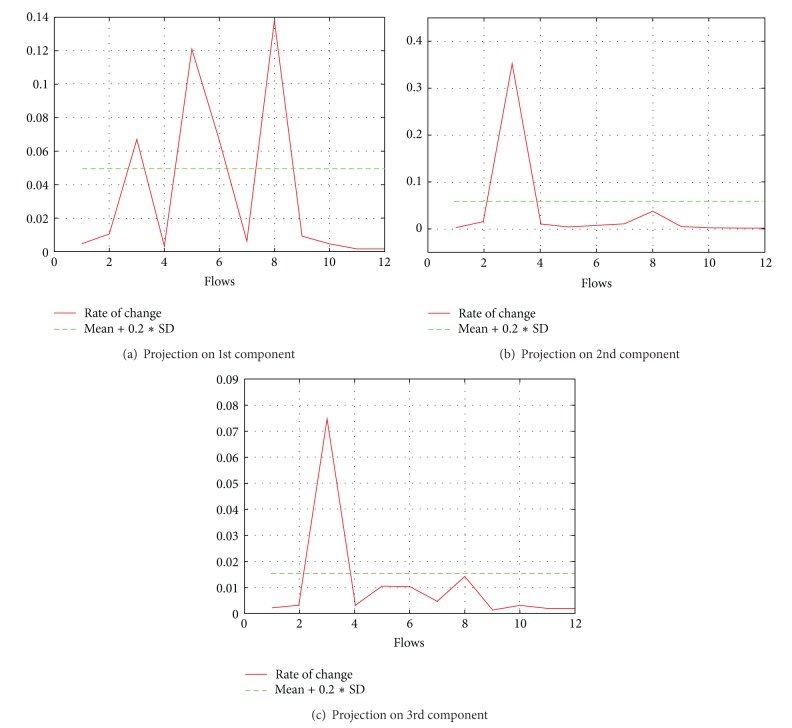
Results of asymmetric topology with RED as drop policy and projecting on different components of diagonal matrix.

**Figure 6:**
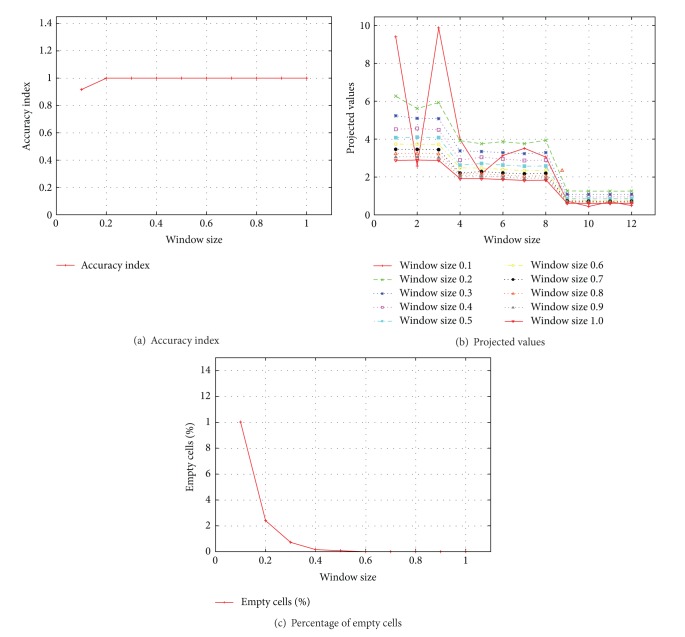
Results of window size experiments.

On the basis of the above observations we propose a general principle to choose a window size for any environment such that it may not yield a percentage of empty cells of more than 2%. In the rest of our experiments, we used a window size of 0.3 which worked fine with all the dynamic network conditions and environments that we simulated. Another strength of our approach revealed in the above experiments was the amount of data required to converge to accurate results. By increasing the window size to 30 seconds we had only 2 average measurements per flow. In such situation, we had a delay matrix of 12 × 2 and it resulted in only 2 values in the diagonal matrix Σ, but even with such a small number of values in the diagonal matrix we had perfect results after the projection step.

(*4) Drop Policy.* We observed some interesting results in simulations using the asymmetric topology with different drop policies like RED and DropTail. DropTail performed well and showed a very promising behavior as shown in Figure 4. However in the case of random early detection (RED), while adopting our usual procedure, we found a small margin of inaccuracy. This inaccuracy occurred because we could not reach the desired rank reduction in the result of SVD. Thus, we projected our data on 2nd and 3rd components, and consequently the results became more accurate as shown in Figure 5.

**Figure 7:**
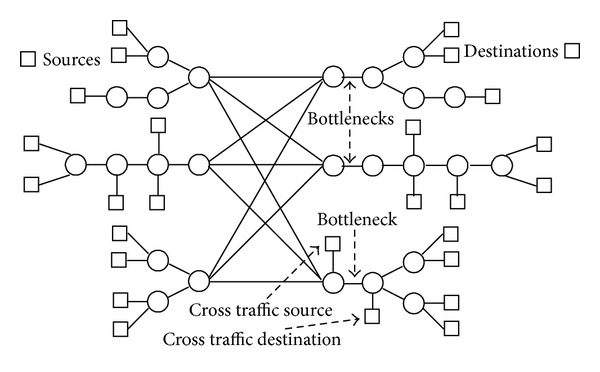
Topology without common endpoints.

It is not surprising that our mechanism has problems with RED, which improves fairness and reduces phase effects by randomly dropping packets [12]—any technique that tries to detect dependencies between flows will naturally suffer from a mechanism that is designed to work against these dependencies. Nevertheless, some dependencies may still remain; thus, although DropTail is commonly known to be the most common drop policy used in routers, we feel that we should work on the automatization of our mechanism to work with all possible drop policies by introducing some additional decision making during the projection phase. This is planned as future work.

(*5) Buffer Size*. Our additional tests included scenarios with different buffer sizes from 50 to 500 packets. The results showed no significant difference. With DropTail, the AI was always 1. We also computed the AI after every 5 seconds for all above scenarios, starting from second 1 of the simulation till its end. We found that the AI value was 1 throughout the simulations.

(*6) Without Common Endpoints*. In our experiments so far, we have used topologies with a common source. It has also been the major assumption in related work to have a single common source or destination. Here, we evaluate our technique for scenarios with multiple sources and destinations. The topology used for these experiments is shown in Figure 7. We have created it by considering the mirror image of all three branches of the asymmetric topology shown in Figure 2(b). The center of the topology represents an overprovisioned wide-area network backbone, whereas bottlenecks are placed at the access points of local networks, which resembles the most common real-life case of Internet connectivity. Two of the three bottlenecks are limited because of their small physical capacity (1.5 Mbps and 3.0 Mbps, resp.) whereas the third bottleneck is caused by heavy cross traffic. The parameters and details for cross traffic and background (forward and reverse) traffic are the same as with previous topologies—these parameters are mentioned in Table 1 except for the nonbottleneck link capacities which were 100 Mbps for this topology.

**Figure 8:**
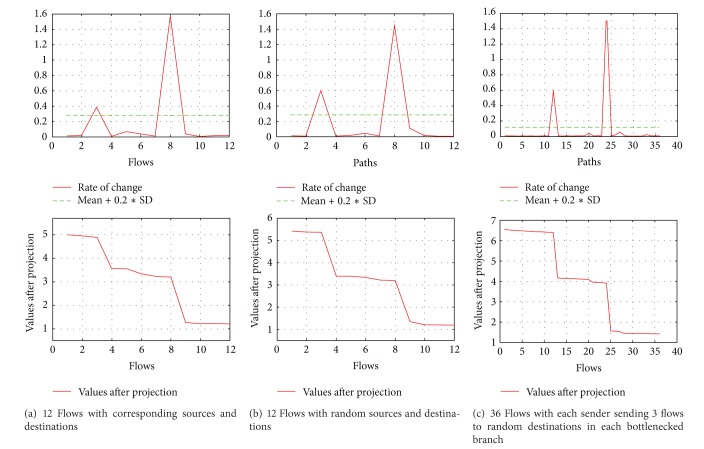
Results of experiments without common endpoints.

In our first experiment we generated 12 TCP flows, one from each source to the corresponding destination in the mirror. We take it as a basic step to show the accuracy of our mechanism which does not need any synchronization between the sending time of senders and does not require packets of different flows to be adjacent, which was required in related work. We randomly start all the flows during the first second of the simulation. After gathering the delay measurements, applying SVD, and then detecting the outliers to form the groups of flows which share a bottleneck, we found the accuracy index (AI) to always be 1. This is also shown in Figure 8(a) which indicates that the first three flows, next five flows, and last four flows share a bottleneck.

As a next step, we again generated 12 TCP flows but from random sources to random destinations; again, our SVD based mechanism resulted in a very nice rank reduction and then clearly showed the outliers to form the group of flows which share a bottleneck. The results are shown in Figure 8(b).

As a final step, we generated three TCP flows from every sender to a randomly selected destination in each branch of the bottleneck. In this way, we generated 36 flows in total, generating foreground traffic from each sender to every branch of the bottleneck. Even with such a heavy foreground and background traffic our mechanism was stable and reliable enough to produce clusters of flows having common bottlenecks with an AI of 1, which is shown in Figure 8(c).

*(7) Fluctuating Bottlenecks. *It is quite possible in real networks that heavy cross traffic may cause a link to behave as a bottleneck for an end-to-end path for a while, and then a routing change or some sudden bursts may cause another link to become the bottleneck. Therefore, in our next experiments, we evaluated the adaptability of our mechanism in case of fluctuating bottlenecks.

**Figure 9:**
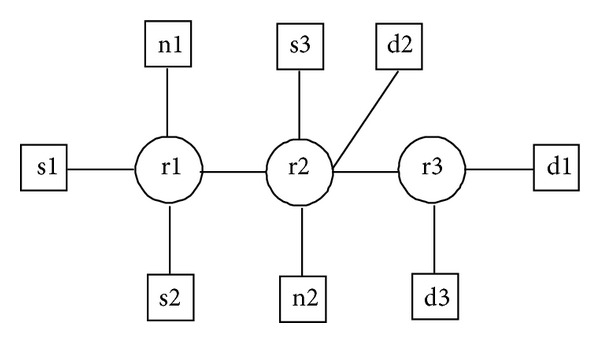
Topology with a varying bottleneck.

The topology used for these experiments is depicted in Figure 9. All the links have a capacity of 10 Mbps, the queue length is 250 packets, and the packet drop policy is DropTail. We generated three TCP flows as foreground flows from senders s1, s2, and s3 to the destinations d1, d2, and d3, respectively, giving us three distinct end-to-end paths p1, p2, and p3. The link between r1 and r2 as well as the link between r2 and r3 may become bottlenecks. If the link between r1 and r2 is behaving as a bottleneck, then the paths p1 and p2 will share a bottleneck; if the link between r2 and r3 is congested, then the paths p1 and p3 will share a bottleneck. Hence, the bottleneck on path p1 may fluctuate from one link to another. We congest the links between r1, r2, and r3 by using a number of CBR flows. In addition to foreground TCP flows and CBR flows as cross traffic, we also started some Pareto flows as background traffic. These Pareto flows are originated from all sources and destinations (including nodes n1 and n2) in both forward and reverse directions. The flows in forward direction were initiated with a constant bit rate of 256 Kbps whereas the flows in reverse direction were sent at a rate of 64 Kbps. The average ON and OFF times were set to 500 ms and the Pareto shape parameter was set to 1.5.

**Figure 10:**
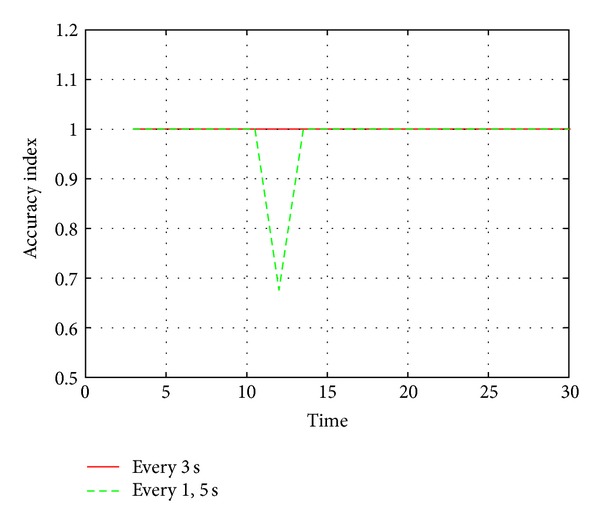
Accuracy index for every 3 seconds and for every 1.5 seconds.

In our simulation of 30 seconds we congested the first link for the first 12 seconds of the simulation and then shifted the heavy cross traffic to the second link. As a result, for the first 12 seconds, paths p1 and p2 shared a bottleneck, and, for the rest of the simulation, paths p1 and p3 shared a bottleneck. Initially we calculated the AI for every 3 seconds and found that it accurately detected the bottleneck shift around the 12th second of the simulation. Then, in order to check the adaptability of our mechanism at a fine grained level, we also calculated the AI for every 1.5 seconds from the 9th second of the simulation till the 15th second of the simulation because this was the time when the bottleneck shifted. This time we noticed a drop in the AI (suggesting an inaccurate clustering) around the 12th second, as shown in Figure 10.

From this experiment we came to know that our mechanism was efficient enough to adopt the shift in the bottleneck within 3 seconds, which is quite acceptable because, if the bottleneck fluctuates every time within less than 3 seconds, then this information will probably not be very useful.

**Figure 11:**
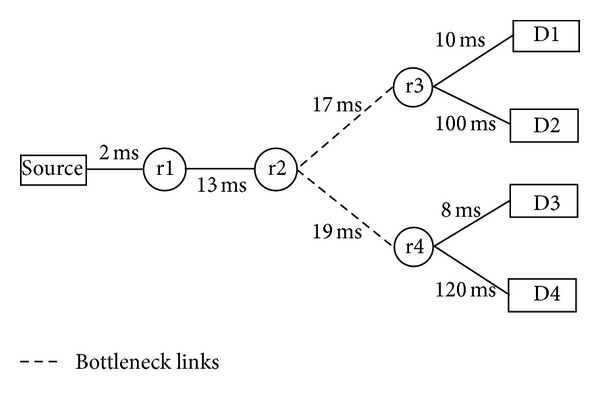
Topology with longer delays at some branches after the bottleneck.

*(8) Topology with Longer Delays after the Bottleneck*. This section highlights a problem that occurs if the delay matrix is used as an input for the shared bottleneck detection algorithm. This problem is not very common but may occur in some special scenarios—this is evident from all the experiments presented so far, where the delay matrix was used as an input for the shared bottleneck detection algorithm and no problem was visible in the results. A scenario where such problem might occur is depicted in Figure 11. In this topology, links from r3 to D2 and from r4 to D4 have delays 100 ms and 120 ms, respectively. These delays make one path after each bottleneck much longer than others in the same branch. All other simulation parameters regarding foreground and background traffic are the same as the ones used in previous experiments. 

Due to this setup, when forward delays are measured from the source to all destinations, then delays from the source to D2 and D4 become similar in terms of their magnitude. These magnitudes overlap so closely that this overlapping outweighs their delay patterns. This can be observed from the projection results presented in Table 3. When the delay matrix was used for shared bottleneck detection, the projection results put path source D4 in first cluster because the forward delay values for this path were highly overlapping with the path source D2. In order to resolve this issue, the covariance matrix is used for shared bottleneck detection. Then, it can be observed from the projection values presented in Table 3 that both clusters were accurately found. 

### 5.3. Comparison with FlowMate

**Figure 12:**
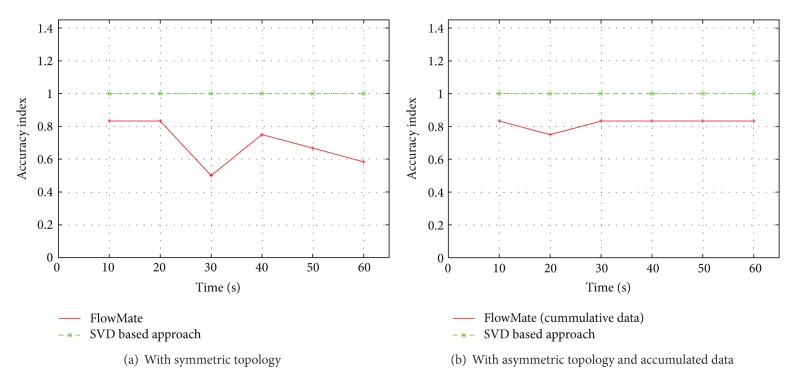
Comparison with FlowMate.

We compared our mechanism with FlowMate which, to the best of our knowledge, is the best of the mechanisms in terms of accuracy and computational complexity.

(*1) Asymmetric Topology*. We used the asymmetric topology illustrated in Figure 2(b) for the comparison. We used 256 Kbps Pareto flows as background traffic from the source to all destinations and 64 Kbps Pareto flows as background traffic in the reverse direction from all destinations to the source. All flows started randomly during the initial phase of the simulation. We used TCP flows as foreground traffic from the source to all destinations to measure the forward delays for all paths during the whole period of simulation, and these TCP flows were also started randomly during the first second of the simulation. As the FlowMate and our SVD based method both require forward delays along the paths for shared bottleneck detection, we used the same data for both methods. We applied the FlowMate algorithm and our SVD based mechanism after every 10 seconds to cluster the flows with shared bottlenecks and calculated the AI. Our mechanism always produced accurate results whereas the results produced by FlowMate were fluctuating under the 100% accuracy line as shown in Figure 12(a). Most of the time the performance of FlowMate was degraded by false splitting; that is, a correct cluster was split into further clusters, and in a few occasions false sharing was also observed. This is shown in Table 4.

(*2) FlowMate with Cumulative Data.* We used the setup from Section 5.3(1) to evaluate and compare FlowMate in a different manner. We decided to apply both mechanisms on accumulated data. The FlowMate is very selective in terms of picking samples to apply the correlation test as it needs adjacent packets for the cross measure *M*_*x*_, and for the automeasure *M*_*a*_ it needs packets separated by a certain value *t*. So the main reason behind this experiment was to observe and compare the performance of FlowMate when it has more than the sufficient amount of data. Our SVD based mechanism was already proven to be good enough to provide accurate results with a small amount of data, and this experiment also showed that our SVD based mechanism can perform well even with a smaller amount of data. We calculated AI after every 10 seconds of the simulation time after applying both mechanisms on accumulated data. FlowMate performed better than before and resulted in improved results as shown in Figure 12(b), but it still could not reach the 100% accuracy level. Another thing observed in this experiment was the nature of errors committed by FlowMate: for accumulated data the errors were only due to false splitting and we did not observe any false sharing.

## 6. Conclusion and Future Work

In this paper we introduced a novel method for detecting shared bottlenecks in the network. By using SVD and applying a scheme for outlier detection on its output, we managed to obtain a perfectly accurate result in almost all of our simulations. The only exceptions from this rule are the obvious case of RED (which works against our mechanism) and the simulation of varying bottlenecks, where we deliberately tried to find the limit of our mechanism's ability to adapt to dynamic network conditions. In the RED simulations, we were able to enhance the precision by projecting the diagonal matrix over second column of the left singular matrix.

Clearly, this high reliability is the main strength of our mechanism. We believe that, combined with its operational simplicity, we may have reached a turning point with our scheme where shared bottleneck detection may no longer be a mere measurement exercise but can in fact become a key practical element of future network or transport layer mechanisms. In addition to extending the performance evaluation of our mechanism with real-life measurements, we intend to contribute to this change in several ways in our future work, where we will embed our shared bottleneck detection method in the transfer delay prediction system that was briefly mentioned in the introduction as an immediate next step.

## Figures and Tables

**Algorithm 1:**
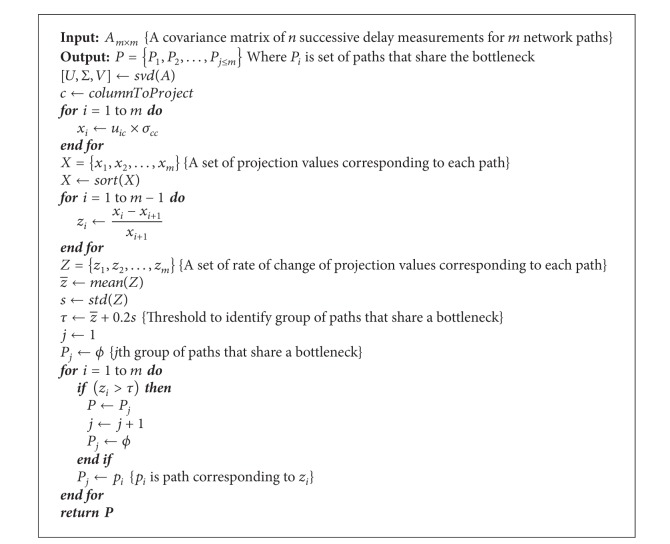
Detection of paths that share a bottleneck.

**Table 1:** Simulation parameters.

Foreground flows	9–27 TCP flows
Background traffic	256 Kbps Pareto flows (source to destinations)
Reverse traffic	64 Kbps Pareto flows (destinations to source)
Cross traffic	64 Kbps CBR flows (24)
Queue size	250 packets (in one experiment 50, 100, 150,… 250)
Drop policy	DropTail (one experiment with RED and RED + DropTail)
Link delays	Randomly assigned (from 1 ms to 20 ms)

**Table 2:** The values of the diagonal matrix and the final result after projection.

Flows	1	2	3	4	5	6	7	8	9
Σ	9.9184	1.1069	0.3380	0.2304	0.1663	0.0645	0.0329	0.0308	0.0251
Projection result	−5.0970	−5.1072	−5.1147	−2.4315	−2.4323	−2.4201	−0.9030	−0.8959	−0.9206

**Table 3:** Projection results with delay matrix and covariance matrix.

Path	Delay matrix	Covariance matrix
*source*-D1	3.2512	0.4687
*source*-D2	4.6151	0.4508
*source*-D3	0.717	0.0027
*source*-D4	2.224	0.0033

**Table 4:** Results of FlowMate and our SVD based approach with three correct clusters ({1, 2, 3}, {4, 5, 6, 7, 8}, and {9, 10, 11, 12}). Our SVD based approach always reported the accurate clusters whereas false sharing and wrong splitting were observed in FlowMate results.

Output clusters	AI	Interpretation
{1, 2}, {3}, {4, 5, 6, 8}, {7}, {9, 10, 11, 12}	0.833	2 errors: false splitting
{1, 2, 3}, {4, 5, 6}, {7, 8}, {9, 10, 11}, {12}	0.833	2 errors: false spitting
{1, 2, 3, 4, 5, 6, 7, 8, 10}, {9, 11, 12}	0.500	6 errors: false sharing
{1, 2, 3, 4}, {5, 6, 11}, {7, 8}, {9, 10, 12}	0.750	3 errors: 2 false splittings and 1 false sharing
{1, 2, 12}, {3, 11}, {4, 5, 6, 7, 8, 10}, {9}	0.667	4 errors: 1 false splitting and 3 false sharings
{1, 2, 10}, {3}, {4, 5, 6}, {7}, {8, 11}, {9, 12}	0.583	5 errors: 3 false splittings and 2 false sharings
